# Cadmium bioaccumulates after acute exposure but has no effect on locomotion or shelter-seeking behaviour in the invasive green shore crab (*Carcinus maenas*)

**DOI:** 10.1093/conphys/cox057

**Published:** 2017-09-28

**Authors:** Tamzin A Blewett, Dustin Newton, Shannon L Flynn, Daniel S Alessi, Greg G Goss, Trevor J Hamilton

**Affiliations:** 1 Department of Biological Science, University of Alberta, Edmonton, Alberta, Canada; 2 Department of Psychology, MacEwan University, Edmonton, Alberta, Canada; 3 Department of Earth and Atmospheric Sciences, University of Alberta, Edmonton, Alberta, Canada; 4 School of Natural and Environmental Sciences, Newcastle University, Newcastle upon Tyne, United Kingdom; 5 Neuroscience and Mental Health Institute, University of Alberta, Edmonton,Alberta, Canada

**Keywords:** Cadmium, neurotoxicity, green shore crabs, open field test, shelter test

## Abstract

Cadmium (Cd^2+^) is a non-essential metal ubiquitous in the environment due to industrial processes. However, little is known regarding the ability of Cd^2+^ to impact the behaviour of aquatic animals in receiving environments. Green shore crabs (*Carcinus maenas*) were exposed to waterborne Cd^2+^ [control (no Cd^2+^), low (0.30 μmol/L), medium (3.3 μmol/L) and high (63 μmol/L)], for 24 h, then, crabs were placed in an open field and shelter test to determine potential changes in locomotion and preference for shelter. Tissues (gill, haemolymph, stomatogastric ganglion) were taken for bioaccumulation analysis of Cd^2+^ and ion content. Behavioural testing was recorded with a motion-tracking software system and showed no impact of Cd^2+^ on any variable in either of the tests used. All three tissues accumulated Cd^2+^ in a concentration-dependent manner. Crabs exposed to low Cd^2+^ showed a small but significant decrease in haemolymph Ca^2+^, however, this effect was not present at higher Cd^2+^ exposures. Overall, the results indicate that short-term Cd^2+^ exposure, and the resulting Cd^2+^ accumulation, had no effect on locomotor and anxiety-related behaviour of green shore crabs.

## Teaser

Cadmium (Cd^2+^) bioaccumulates in tissue in a dose-dependent manner after a 24 h exposure in green shore crabs (*Carcinus maenas*), but this has no impact on basic behaviour.

## Introduction

Cadmium (Cd^2+^) is a non-essential metal with a wide range of industrial uses, including the production of batteries, pigments, stabilizers and some alloys (ICA, 2000). Naturally occurring Cd^2+^ can also be enriched through processes such as mining and through the manufacture of fertilizers (Chandurvelan, *et al.*, 2012). Consequently, processes like smelting, mining and agricultural run-off can lead to enhanced concentrations of Cd^2+^ in aquatic settings (McGeer *et al.*, 2012). In relatively uncontaminated freshwater, dissolved Cd^2+^ is usually <0.004 μmol/L, while levels in open seawater are ~0.0017 μmol/L (Pan *et al.*, 2010). However, in contaminated waters Cd^2+^ concentrations can be as high as 7.1 μmol/L (Sabti *et al.*, 2000; Craw *et al.*, 2005), with sediment values up to 3.5 mmol/kg (Lalor, 2008). In general, estuarine and marine coastal aquatic environments are considered sinks for metals coming from both natural and anthropogenic sources (Henry *et al.*, 2012).

The presence of Cd^2+^ in estuarine settings threatens the health of organisms living in these environments. For example, marine invertebrates may take up Cd^2+^ from the water column across epithelia in contact with the external milieu. For crustaceans, Cd^2+^ uptake is likely to occur via the gills. The crab gill consists of a single layer of epithelial cells (Compere *et al.*, 1989), resulting in a very thin barrier between the haemolymph and the external environment (Henry *et al.*, 2012). While ideal for gas/nutrient exchange, these characteristics also facilitate metal accumulation and transport. Free Cd^2+^ ions have similar ionic radii (0.92 Å) to those of Ca^2+^ (0.94 Å) (Williams and Frausto da Silva, 1996), and as such, Cd^2+^ moves across the apical epithelium via Ca^2+^ channels (Verbost *et al.*, 1989; Lucu and Obersnel, 1996; Pedersen and Bjerregaard, 2000; Henry *et al.*, 2012; Ortega *et al.*, 2014). It has also been shown that Cd^2+^ uptake can be blocked with the Ca^2+^ channel blockers lanthanum (Lucu and Obersnel, 1996; Borowitz and McLaughlin, 1992; Pedersen and Bjerregaard, 1995), and verapamil (Ortega *et al.*, 2014).

Once absorbed, even low concentrations of Cd^2+^ can cause toxicity to aquatic biota. Reported toxic effects of Cd^2+^ include respiratory impairment in fish at high levels (>1000 mmol/L) (Carpenter, 1927) and alteration of ion homoeostasis, most notably Na^+^, Mg^2+^ and Ca^2+^ (Pratap *et al.*, 1989). However, based on its mimicry of Ca^2+^ and inhibition of the important branchial Ca^2+^ transporter Ca^2+^-ATPase, the most prominent effect of waterborne Cd^2+^ is disruption of Ca^2+^ homoeostasis (Wood, 2001; Niyogi and Wood, 2004).

While the uptake and toxicity of Cd^2+^ to model aquatic species, such as the green shore crab (*Carcinus maenas*), has been studied in some detail (Wright, 1977; Wright and Brewer, 1979; Wicklund and Runn, 1988; Bjerregaard and Depledge, 1994), there has been very little investigation of the effects of Cd^2+^ on behaviour in this, and other, estuarine species. However, previous literature has shown that in freshwater amphipods, Cd^2+^ increases mobility, decreases refuge use, and alters the response to alarm stimuli (Sornom *et al.*, 2012). In marine species like squid, Cd^2+^ impacts locomotion which is mediated by the Cd^2+^-induced blockade of Ca^2+^ currents in neurons (Chow, 1991). In fiddler crab larvae (*Uca pugilator*) and shrimp (*Palaemonetes pugio*) exposed to Cd^2+^ locomotion, activities were suppressed which was attributed to a reduced metabolic rate (Vernberg *et al.*, 1974; Hutchenson *et al.*, 1985) resulting from decreased functional lamellar area of the gills impairing energy production and oxygen uptake (Xuan *et al.*, 2014). In other marine shrimp species (*Hippolyte inermis*) Cd^2+^ reduced swimming velocity (Untersteiner *et al.*, 2005). These studies suggest that Cd^2+^ exerts toxic effects on aquatic biota via impairment of behaviour, but knowledge of the mechanisms of behavioural toxicity, and how these may be conserved among biota, is not well understood.

Decapod crustaceans demonstrate complex behaviours which can be interpreted from their retreat from noxious stimuli. Multiple studies have shown that a small electric shock causes retreat from desirable shells in hermit crabs (Elwood and Appel, 2009) and this avoidance learning can persist for up to 24 h (Elwood and Appel, 2009). Noxious stimuli will elevate stress, which has been shown in crayfish to be mediated by serotonin, and which alters anxiety-like behaviour in an aquatic light/dark plus maze (Fossat *et al.*, 2014). Therefore, regulation of stress by neurotransmitters and hormones can determine whether a decapod will change its behaviour, including those associated with anxiety-like responses and search for shelter. For example, acute exposure to a known antidepressant, fluoxetine (Prozac), decreases time spent in the dark zone of a light/dark arena, indicative of decreased anxiety (Hamilton *et al.*, 2016). *Carcinus maenas* naturally prefer a dark shelter compared to a well-lit environment (Barr and Elwood, 2011), based on their natural tendency to hide under rocks and in crevasses in intertidal regions. This characteristic has been used to demonstrate the presence of discrimination learning in this species of crab. When administered an electric shock in one shelter they can learn to discriminate and choose to retreat to a second shelter they were not previously shocked in Magee and Elwood (2013). Taken together, *C. maenas* are an ideal species to study the potential changes in anxiety-like behaviour and tendency to seek shelter after exposure to toxicants that may bioaccumulate.

The current study aimed to examine the concentration-dependent effects of Cd^2+^ on behaviour of the model estuarine species, *C. maenas*. This species inhabits near-shore estuarine and marine settings that are likely to be subjected to impacts from anthropogenic Cd^2+^ contamination (Rodrigues and Pardal, 2014). Previous studies have shown that this species is sensitive to metals, with sub-lethal impacts of the trace metal Ni^2+^ apparent at environmentally relevant concentrations (Blewett *et al.*, 2015; Blewett and Wood, 2015). This animal is also a key model in ecotoxicity testing (Leignel *et al.*, 2014; Rodrigues and Pardal, 2014). Cd^2+^ has been previously shown to exert toxicity in *Carcinus* through mechanisms associated with Ca^2+^ disruption (Bjerregaard and Visilie, 1985; Burke *et al.*, 2003). In the current study we assessed the effects of Cd^2+^ at a range of concentrations from low environmental (~0.3 μmol/L) to supra-environmental (~63 μmol/L), on tissue Cd^2+^ accumulation (gill, neuronal, haemolymph), haemolymph ion content and behaviour. Behaviour was assessed with two tests given in succession: the open field test to examine locomotion and thigmotaxis (a proxy for anxiety-like behaviour), and a shelter test to examine the tendency to move towards a previously experienced shelter. This novel approach will identify whether behavioural changes could be used as a non-lethal indicator of Cd^2+^ toxicity. Behavioural effects of Cd^2+^ were examined over acute, 24-h exposures. Owing to the near-shore tidally influenced habitats most susceptible to Cd^2+^ contamination, crabs may be expected to experience relative high pulsed exposures of metals over relatively short time-frames (Handy, 1994). Our study was designed to replicate such an exposure scenario.

## Methods

### Animal collection

Male green shore crabs (*C. maenas*) (*n* = 80; mean mass = 13.5 ± 0.7 g; mean carapace width = 3.9 ± 0.1 cm) were wild caught via baited traps under a license from Fisheries and Oceans Canada. The collection area was an uncontaminated site (N49°0.274–W125°20.710) located on the west coast of Vancouver Island, British Columbia, Canada. Crabs were collected at 32 ppt and 13°C. Following capture, crabs were transported to Bamfield Marine Sciences Centre (Bamfield, British Columbia, Canada) and were allowed to acclimate for one week prior to experimentation. During this period, crabs were housed in large 200-L outdoor tanks, containing PVC pipe for shelter, and supplied with flow-through seawater (32 ppt) at 13°C under constant aeration. Crabs were fed to satiation every three days on a diet of salmon heads, but were fasted for 48 h prior to experimentation. All research followed Canadian Council on Animal Care standards for aquatic animals.

### Cadmium exposure

Crabs were randomly distributed into one of four experimental groups with two replicates per treatment: control (0 μmol/L Cd^2+^; *n* = 20), low (0.3 μmol/L Cd^2+^; *n* = 20), medium (3.3 μmol/L Cd^2+^; *n* = 20) and high (63 μmol/L Cd^2+^; *n* = 20) (measured values; reported in Table 1). Cd^2+^ exposures were conducted in 10-L plastic containers (10 L) with constantly aerated seawater (in mM: Na, 492; K, 9; Ca, 12; Mg, 50; Cl, 539; pH 8.0; Blewett *et al.*, 2015), at the appropriate Cd^2+^ dilution. Exposure chambers were placed in a recirculating water table to keep water temperature constant (13°C), and matched to both collection and holding temperature. Cd^2+^ was added from a concentrated stock (CdCl_2_·6H_2_O; Sigma Aldrich), 24-h in advance of experimentation to allow for the metal to reach chemical equilibrium. After 24-h of exposure crabs underwent behavioural testing.

**Table 1:** Water Chemistry of exposure tanks taken at 0 and 24 h after exposure. ND = not detectable (*n* = 24)

Condition	Control (0 Cd)	Low	Medium	High
Measured Cd (μmol/L)	ND	0.30 ± 0.0001	3.3 ± 0.07	63 ± 0.5
Temperature (°C)	13	13	13	13
Salinity (ppt)	33 ± 0.3	34 ± 0.8	33.1 ± 0.2	34.2 ± 0.3

### Behavioural testing

The exposure chamber containing the crabs was moved to the testing facility 10 min prior to the first trial to minimize any potential stress due to transportation. Crabs were individually moved by hand from the exposure container to the open field testing arena, which was a white plastic circular arena (69 cm diameter, 25 cm walls). Seawater of composition identical to that of the exposure, but without Cd^2+^, was filled to a depth of 8 cm prior to the testing of each exposure group. The order of testing of Cd^2+^ groups was reversed for each replicate. Individual crabs were placed in the centre of the arena and were recorded with motion-tracking software (Ethovision XT, v.10; Noldus Information Technology, Leesburg, VA, USA). All open field trials were 5 min in duration and began immediately when crabs were placed into the arena. At the end of the trial the novel object/shelter was placed into the arena in the same location against the wall of the arena and the crab was gently moved to the opposite side of the arena with a plastic metre stick. The shelter was a PVC pipe (10 cm diameter, 8.5 cm long) that was weighted down to prevent movement. This was used as a shelter because all crabs were previously exposed to these PVC pipes when habituated upon arrival to the field station and PVC pipes were also present in the exposure containers.

All testing trials were 5 min in duration, and data were recorded and analyzed with Ethovision XT. Distance moved (cm), immobility (s) and time in zones (s) were quantified in Ethovision XT. In the open field test we quantified time in the inner zone (a circle of 48 cm diameter centred in the middle of the arena) as this is a measure of exploration and a lack of time in the outer zone (thigmotaxis zone) is a common proxy for decreased anxiety-like behaviour. In the shelter test we quantified the time spent in the shelter zone (a circle of 34.5 cm centred on top of the shelter) and average distance to the shelter.

### Tissue/water sampling

Immediately after behavioural testing, crabs were placed on ice (15 min) before terminal sampling. A 1-mL haemolymph sample was collected via a puncture at the bases of the third and fourth pair of periopods. Crabs were then quickly euthanized by single puncture to the ventral ganglion through the carapace, before being washed in 1 mM EDTA for 1 min to remove any adsorbed Cd^2+^ (Blewett *et al.*, 2015; Blewett and Wood, 2015). Gill pair 8, and the stomatogastric ganglion were then taken, weighed, and all tissues (including haemolymph) were digested in 2 N trace metal grade nitric acid (Sigma Aldrich) at volumes three to five times the weight of the tissue. Tissues were digested at 65°C for 48 h with vigorous vortexing at 24 h. After 48 h the digested tissue was then centrifuged for 5 min at 4000 rpm at 18°C. Volumes of 800 μL were then taken for analysis of Ca^2+^ ion content (haemolymph) and Cd^2+^ (all tissues) as detailed below.

### Water and tissue analyses

Exposure water concentrations were monitored at *t*= 0 and 24-h in each chamber (*n* = 24 per concentration). There was no statistical difference in measured exposure Cd^2+^ concentration between 0 and 24 h and thus average values are reported in Table 1. Water samples for Cd^2+^ analysis were filtered through a 0.45 μm membrane (Acrodisc; Pall Life Sciences, Houston, TX, USA). There was less than a 5% difference between filtered and unfiltered samples and therefore only dissolved Cd^2+^ is shown in Table 1.

Tissue (Gill 8, haemolymph and stomatogastric neuron) samples were further diluted and acidified using 2% trace metal grade nitric acid and 0.5% trace metal grade nitric acid at a ratio of Ca^2+^ and Cd^2+^. All analyses were performed using an Agilent 8800 Triple Quadrupole ICP-MS (ICP-MS/MS) with an RF power of 1550 W and a RF reflected power of 18 W. The ICP-MS/MS was operated with a microMist nebulizer and nickel/copper cones tested against Environment Canada Certified reference materials. Several unique features of the ICP-MS/MS were utilized during analysis including MS/MS mode for greater mass resolution and the collision/reaction cell with H_2_ gas. Additionally, an inline internal standard system was employed with a solution of 0.5 mg/L indium which was used to correct for instrumental drift. Standards were made in a matrix of 2% trace metal grade nitric acid and 0.5% trace metal grade nitric acid covering a range of 0.001–25 mg/L. Replicate standards were found to vary by ±4.9% through the duration of a run. All standard curves had *R*^2^ > 0.98. All tissue concentrations were defined per gram of tissue.

### Statistical methods

Normality was tested via the D’Agostino and Pearson omnibus test. One-way ANOVAs were used for parametric data, and Kruskal–Wallis tests with Dunn’s multiple comparison post hoc tests were used for data that were not normally distributed. All accumulation and haemolymph data were analyzed by a one-way ANOVA using a Tukey post hoc analysis. An *α*-level of 0.05 and 95% confidence intervals were used for assessing statistical significance in all tests. We used *t*-tests and Mann–Whitney tests to compare the two replicate groups per treatment condition. Non-significant differences allowed us to combine groups. There were significant differences in the open field test between the replicates in the Medium (3.3 μmol/L) group for distance moved, (*P* = 0.0211, *t*= 2.526, df = 18, unpaired *t*-test) and immobility, (*P* = 0.045, *t *= 2.154, df = 18, unpaired *t*-test). Similarly, in the novel object/shelter test a significant difference was also observed in the low (0.3 μmol/L) replicate groups for distance moved (*P* = 0.028, *t* = 2.390, df = 18, unpaired *t*-test). However, in every case neither replicate was different from the control group so these data were still pooled. Behavioural data were analyzed using GraphPad Prism (v.5, San Diego, CA, USA). Bioaccumulation significance was tested via SigmaPlot 11 (Systat software Inc., San Jose, CA, USA) and Sigma Stat 3.5 (Systat Software Inc., San Jose, CA, USA) using a one-way ANOVA and a Tukey post hoc test. All data are presented as mean ± SEM.

## Results

### Water Chemistry

In control exposures, Cd^2+^ was below detectable limits for the ICP-MS/MS (0.11 μmol/L). Cd^2+^ exposure values were 0.30 ± 0.00 μmol/L in our low exposure, 3.3 ± 0.1 μmol/L in the medium exposure group, and 63 ± 1 μmol/L in the high exposure treatment group (Table 1).

### Tissue cation measurements

**Figure 1:**
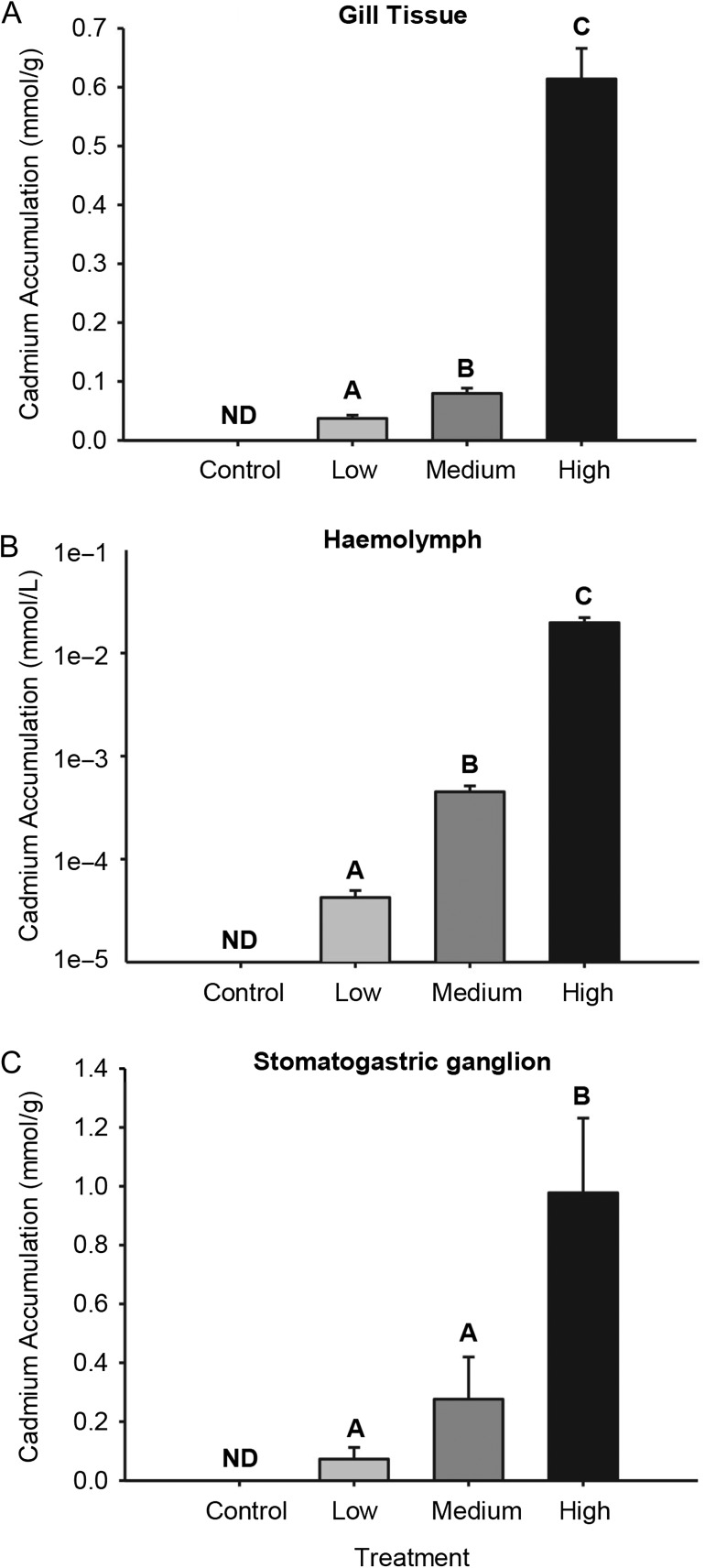
Cd^2+^ accumulation in tissues. (**A**) Gill 8; (**B**) haemolymph; (**C**) stomatogastric ganglion of green shore crab (*Carcinus maenas*) after a 24-h exposure to one of four different treatment groups [control (no added Cd^2+^), low (0.3 μmol/L), medium (3.3 μmol/L) and high (63 μmol/L)]. Means sharing the same letter are not significantly different (*α* = 0.05). Values are means ± SEM (*N* = 19–20 per treatment).

Accumulation of Cd^2+^ increased significantly in all tissues (Gill 8, haemolymph and stomatogastric ganglion) as the exposure concentration increased [gill: *P* < 0.001, *F*(2, 53) = 166.455, one-way ANOVA; haemolymph: *P* < 0.001, *H*(2, 57) = 48.89 Kruskal–Wallis test; stomatogastric ganglion: *P* < 0.001, *H*(2, 57) = 25.8 Kruskal–Wallis test]. The sole exception was in the neuronal tissue, where there was no significant difference in accumulation between low (0.3 μmol/L) and medium (3.3 μmol/L) treatment groups (Fig. 1C). The highest accumulation occurred in the stomatogastric ganglion, which in the highest exposure group accumulated 0.9 mmol Cd^2+^/g (Fig. 1A–C).

**Figure 2:**
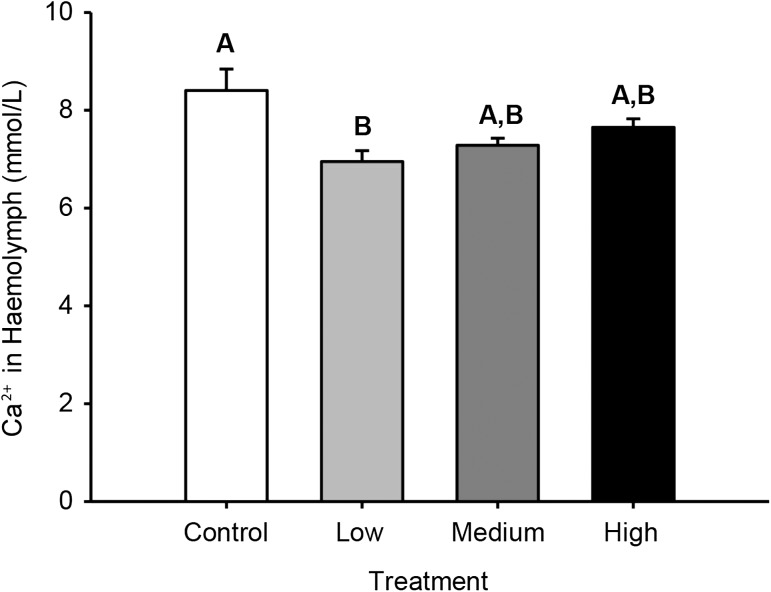
Ca^2+^ concentrations (mmol/L) in haemolymph of green shore crabs (*Carcinus maenas*) after a 24-h exposure to one of four different treatment groups [control (no added Cd^2+^), low (0.3 μmol/L), medium (3.3 μmol/L) and high (63 μmol/L)]. Means sharing the same letter are not significantly different (*α* = 0.05). Values are means ± SEM (*N* = 19–20 per treatment).

Haemolymph Ca^2+^ was significantly decreased [*P* = 0.035, *F*(2, 54) = 170.96, one-way ANOVA] by Cd^2+^ exposure, but only in the low exposure group (0.3 μmol/L). All other exposure groups were not significantly different from control (Fig. 2).

### Open field test

**Figure 3:**
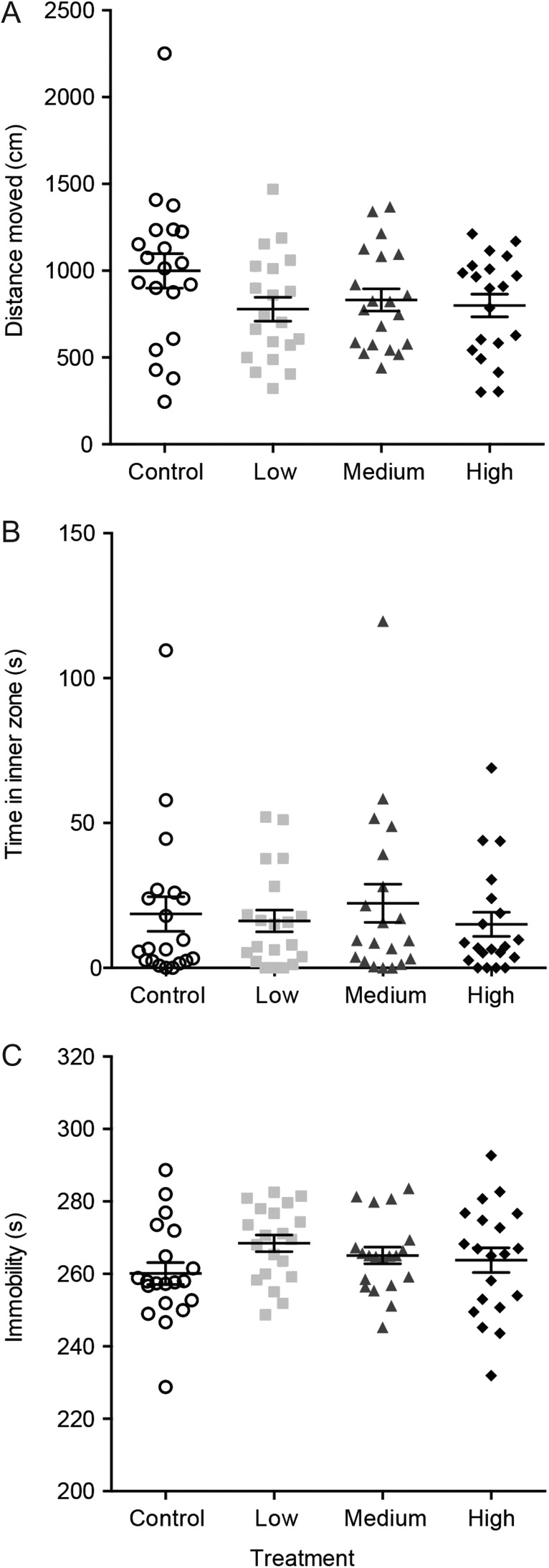
Crab behaviour in the open field test after a 24-h exposure to one of four different treatment groups [control (no added Cd^2+^), low (0.3 μmol/L), medium (3.3 μmol/L) and high (63 μmol/L)]. There were no significant differences in distance moved (**A**), time in the inner zone (**B**) and immobility (**C**). Values are means ± SEM (*N* = 20 per treatment) and symbols are data points for individual crabs.

Green shore crabs exposed to Cd^2+^ showed no significant difference in distance moved [Fig. 3A, *P* = 0.1644, *F*(3, 76) = 1.748, one-way ANOVA] compared to controls. There was no difference in time spent in the inner zone of the arena between Cd^2+^ exposed crabs and controls [Fig. 3B, *P* = 0.9336, *H*(3, 76) = 0.4316, Kruskal–Wallis test]. Immobility was not significantly altered by any concentration of Cd^2+^ exposure compared to controls [Fig. 3C, *P* = 0.2193, *F*(3, 76) = 1.508, one-way ANOVA].

### Shelter test

**Figure 4:**
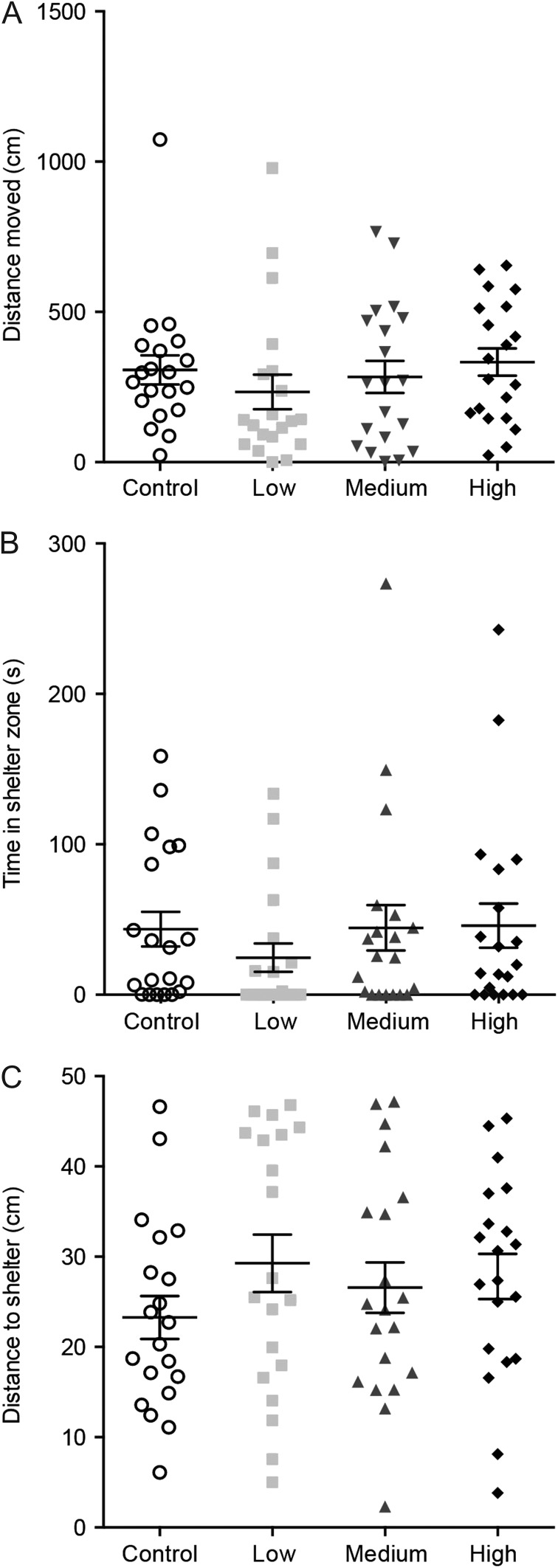
Crab behaviour in the shelter test after a 24-h exposure to one of four different treatment groups [control (no added Cd^2+^), low (0.3 μmol/L), medium (3.3 μmol/L) and high (63 μmol/L)]. There were no significant differences in distance moved (**A**), time in the shelter zone (**B**) and distance to shelter (**C**). Values are means ± SEM (*N* = 20 per treatment) and symbols are data points for individual crabs.

Green shore crabs exposed to Cd^2+^ showed no significant difference in distance moved [Fig. 4A, *P* = 0.1983, *H*(3,76) = 4.661, Kruskal–Wallis test] compared to controls. There was no difference in time spent in the shelter zone of the arena between Cd^2+^ exposed crabs and controls [Fig. 4B, *P* = 0.3030, *H*(3,76) = 3.641, Kruskal–Wallis test]. Distance to shelter was not significantly altered by any concentration of Cd^2+^ exposure compared to controls [Fig. 4C, *P* = 0.4364, *H*(3,76) = 2.722, Kruskal–Wallis test].

## Discussion

After 24 h of exposure, Cd^2+^ accumulated in gill, haemolymph and the stomatogastric ganglion. No Cd^2+^ was detected in control tissues, while in other exposure concentrations the accumulation reflected the dose (Fig. 1A–C). Previous work on crustaceans has shown that during a waterborne exposure, the gills are a target of metal entry (Soegianto *et al.*, 1999). While *Carcinus* is capable of absorbing Cd^2+^ via the gastrointestinal tract (Bjerregaard *et al.*, 2005), the fact that crabs were not fed during exposures and were in osmotic balance and therefore do not drink, makes this an unlikely route of uptake.

Green shore crabs have eight gill pairs with specialized adaptations for different processes. The first four gill pairs are characterized as respiratory in function and the last four pairs are considered to represent the principal site of ion transport. In the current study, Gill 8 was taken for Cd^2+^ accumulation analysis, as previous work has shown that ionoregulatory gills accumulate relatively more metal than respiratory gills (Blewett *et al.*, 2015; Blewett and Wood, 2015). Consequently, the accumulation of Cd^2+^ in Gill 8 in the current study is likely to due to the ability of Cd^2+^ to be taken up via Ca^2+^ transport pathways. In crustaceans, Cd^2+^ enters the gill via voltage–independent non-selective Ca^2+^ channels (Bjerregaard and Depledge, 1994; Pedersen and Bjerregaard, 1995; Ahearn *et al.*, 2001), driven by electrochemical gradients. Cd^2+^ is then likely transported into the haemolymph via the basolateral transporters Ca^2+^-ATPase and/or Na^+^/Ca^2+^ exchanger (Flik *et al.*,1994). Previous work has confirmed these are the likely transporters, as Cd^2+^ entry can be blocked by Ca^2+^ channel inhibitors such as lanthanum (Pedersen and Bjerregaard, 1995; Lucu and Obersnel, 1996) and diltiazem (Borowitz and McLaughlin, 1992). Once in the haemolymph, Cd^2+^ is principally bound to plasma proteins such as haemocyanin—the major respiratory pigment found in crustaceans (Borowitz and McLaughlin, 1992; Lucu and Obersnel, 1996; Pedersen and Bjerregaard, 1995).

Once absorbed, Cd^2+^ is principally transported to the hepatopancreas of crabs, specifically to R and E cells which are thought to have higher Ca^2+^ demands, and thus greater potential for Cd^2+^ uptake (Ortega *et al.*, 2017). In the hepatopancreas, high concentrations of metal-binding proteins such as metallothionein act to sequester the Cd^2+^, minimizing its ability to interfere with cellular functions (Henry *et al.*, 2012). However, in the current study Cd^2+^ was detected in the stomatogastric ganglion (Fig. 1C). This is a series of interconnected neurons with similar physiological functioning to that of the mammalian brain (Harris-Warrick *et al.*, 1992). The presence of Cd^2+^ in the stomatogastric ganglion therefore suggests a spillover of Cd^2+^ to sensitive tissues. Accumulation of Cd^2+^ in neural tissues may be a consequence of their high concentration of Ca^2+^ channels, facilitating accumulation by mimicry (Bjerregaard and Depledge, 1994).

Previous studies have shown an ability of Cd^2+^ to impact Ca^2+^ homoeostasis after Cd^2+^ absorption. For example, Cd^2+^ has been shown to decrease Ca^2+^ in haemolymph in the amphipod (*Gammarus pulex)* (Felten *et al.*, 2008) and alter gill Ca^2+^-ATPase activity in the grapsid crab (*Chasmagnathus granulata*) (Vitale *et al.*, 1999). In the current study, Cd^2+^ was shown to decrease haemolymph Ca^2+^, consistent with previous studies. However, this effect did not persist with higher Cd^2+^ exposure concentrations, so its toxicological relevance is unknown.

Given the accumulation of Cd^2+^ in nervous tissue, an effect of Cd^2+^ on crab behaviour was hypothesized. However, we observed no effect of Cd^2+^ at any exposure concentration on any of the behavioural endpoints monitored. The open field test is commonly used to monitor locomotor deficits (Heredia *et al.*, 2014). We observed no change in total distance moved and immobility, both locomotion parameters. The open field test can also be used to investigate anxiety-like behaviour, with anxious animals typically spending more time near the walls of the arena (i.e. thigmotaxis) and less anxious animals exploring the centre of the arena. Modulation of anxiety-like behaviour has been recently observed in striped shore crabs (*Pachygrapsus crassipes*) with acute fluoxetine exposure, which results in increased time spent in a light zone of a light/dark test, indicating a decrease in anxiety (Hamilton *et al.*, 2016). Crayfish (*Procambarus clarkii*) also have a natural tendency to seek out the dark zone of a light/dark arena and this can be increased with aversive electrical stressors (Fossat *et al.*, 2014, 2015). In this study we also quantified the time crabs spent near the centre of the arena (inner zone) in the open field test as a proxy for decreased anxiety-like behaviour. Again, we found no differences across all Cd^2+^ exposures suggesting Cd^2+^ had no impact on the neural circuitry regulating anxiety-like behaviour. Secondly, we used a shelter test to examine whether Cd^2+^ exposures may alter the tendency of crabs to seek out an area of refuge, which is already an established behaviour in this species (Barr and Elwood, 2011; Magee and Elwood, 2013). We quantified the time the crabs took to reach the shelter and the average distance to the shelter and found no effect of Cd^2+^ exposure.

The lack of behavioural impacts of Cd^2+^ may be explained by a number of factors. Accumulated Cd^2+^ may not necessarily translate to bioactive Cd^2+^. A previous study has shown that the expression of metallothionein in nervous tissue of the lobster *Panulirus argus* is highly inducible by Cd^2+^, with the levels of induction significantly greater than those observed in other tested tissues (Molto *et al.*, 2005). This may suggest that despite accumulation, the Cd^2+^ present in the stomatogastric ganglion was rapidly bound by metallothionein, and thus not biologically active. If this induction response is significantly more pronounced than that found in other species where effects of Cd^2+^ on behaviour have been shown (Sornom *et al.*, 2012; Untersteiner *et al.*, 2005), then this could explain the relative lack of impact of the accumulated Cd^2+^.

The length of exposure may also be an important factor, as toxicity can take some time to manifest. For example, in a study that compared the physiological responses of mussels to waterborne Cd^2+^, effects on respiration rate were not found after acute exposures (96 h), but were present after 120 h (Chandurvelan *et al.*, 2012). This was despite no significant change in digestive gland Cd^2+^ burden. It is therefore possible that exposures longer than the 24-h period in the current study may lead to behavioural effects. It is, however, worth noting that a previous study in freshwater amphipods observed impacts of Cd^2+^ on behaviour after only a 4-min exposure period (Sornom *et al.*, 2012). Furthermore, electrophysiological studies that apply Cd^2+^ to rodent brain tissue observe effects in tens of minutes (Hamilton *et al.*, 2010). However, it is important to note that the short-term effects of Cd^2+^ were observed in media with higher Cd^2+^ bioavailability (freshwater vs. seawater), at higher exposure concentrations (4.4 μmol/L), and in much smaller animals (15 mm length) (Sornom *et al.*, 2012), making previous work difficult to compare to ours.

A lack of impact of Cd^2+^ on locomotion, also suggests a lack of effect on muscle function. Impairment of movement can manifest through either a behavioural impediment or a physical incapacity to enact the behaviour. Previous studies have shown that a 24 h exposure to Cd^2+^ does alter muscle biochemistry, with an increase in lactate dehydrogenase activity observed in fiddler crabs in response to 2 mg/L (18 μmol/L; Devi *et al.*, 1993). In the current study, despite a higher exposure concentration over a similar exposure duration, no effects on locomotion could be observed. This suggests that even if effects on muscle biochemistry were enacted by Cd^2+^ exposure, these did not translate to measurable impacts on behaviour.

Future research should investigate how these results are applicable to the wild, and whether other marine organisms that cohabit ecosystems with green shore crabs are differentially affected by acute Cd^2+^ exposures. Would environmental exposures to Cd^2+^ be a relatively advantageous situation for the already invasive green shore crab? Would green shore crabs and other organisms move away from an area of high Cd^2+^ exposures, or might only the green shore crabs remain? These questions should be answered in order to attempt to predict the ecological consequences of Cd^2+^ pollution in the oceans and to better understand the resilience of the invasive green shore crab.

It is known that green shore crabs are highly robust animals, capable of handling significant fluctuations in salinity, temperature and oxygen (Henry *et al.*, 2012). In fact, due to their abilities to adapt and survive a wide variety of environmental conditions, *Carcinus* is a successful invasive species. This study demonstrates that they are also tolerant to high levels of Cd^2+^ over acute exposure periods in terms of a lack of impact upon ecologically important behaviourally endpoints, and this resilience is likely to contribute to their future ecological success with increasing environmental pressures.
